# Cellular aspartyl proteases promote the unconventional secretion of biologically active HIV-1 matrix protein p17

**DOI:** 10.1038/srep38027

**Published:** 2016-12-01

**Authors:** Francesca Caccuri, Maria Luisa Iaria, Federica Campilongo, Kristen Varney, Alessandro Rossi, Stefania Mitola, Silvia Schiarea, Antonella Bugatti, Pietro Mazzuca, Cinzia Giagulli, Simona Fiorentini, Wuyuan Lu, Mario Salmona, Arnaldo Caruso

**Affiliations:** 1 Department of Molecular and Translational Medicine, University of Brescia Medical School, Brescia, Italy; 2 Department of Biochemistry and Molecular Biology, University of Maryland, Baltimore, Maryland, USA; 3 IRCCS Istituto di Ricerche Farmacologiche “Mario Negri” Milan, Italy; 4 Institute of Human Virology, University of Maryland, Baltimore, Maryland, USA

## Abstract

The human immune deficiency virus type 1 (HIV-1) matrix protein p17 (p17), although devoid of a signal sequence, is released by infected cells and detected in blood and in different organs and tissues even in HIV-1-infected patients undergoing successful combined antiretroviral therapy (cART). Extracellularly, p17 deregulates the function of different cells involved in AIDS pathogenesis. The mechanism of p17 secretion, particularly during HIV-1 latency, still remains to be elucidated. A recent study showed that HIV-1-infected cells can produce *Gag* without spreading infection in a model of viral latency. Here we show that in *Gag*-expressing cells, secretion of biologically active p17 takes place at the plasma membrane and occurs following its interaction with phosphatidylinositol-(4,5)-bisphosphate and its subsequent cleavage from the precursor *Gag* (Pr55^*Gag*^) operated by cellular aspartyl proteases. These enzymes operate a more complex *Gag* polypeptide proteolysis than the HIV-1 protease, thus hypothetically generating slightly truncated or elongated p17s in their C-terminus. A 17 C-terminal residues excised p17 was found to be structurally and functionally identical to the full-length p17 demonstrating that the final C-terminal region of p17 is irrelevant for the protein’s biological activity. These findings offer new opportunities to identify treatment strategies for inhibiting p17 release in the extracellular microenvironment.

The human immunodeficiency virus type 1 (HIV-1) matrix protein p17 (p17) is a small (17 kDa) basic protein that is cleaved from the precursor *Gag* (Pr55^*Gag*^) by the viral protease during particle release from the cell^1^. The three-dimensional structure of p17 has been determined by nuclear magnetic resonance (NMR) and X-ray crystallography. Individual folded p17 molecules are composed of five major α-helixes and a highly basic platform consisting of three β-strands^2^,^3^. This partially globular protein presents four helixes centrally organized to form a compact globular domain capped by the β-sheets. The fifth helix (H5) in the C-terminus of the protein projects away from the packed bundle of helixes and, according to NMR, p17 conformation in this region may be partially unfolded^4^. The NH_2_-terminal region of p17 is modified by myristoylation, which is important for membrane binding^5^,^6^,^7^. In addition, the p17 highly basic region, which is comprised of residues 17 to 31, interacts with acidic phospholipids in the inner leaflet of the membrane and is required for targeting of *Gag* to the plasma membrane during viral assembly^8^,^9^. The ability of *Gag*, through p17, to co-localize at specific subcellular membranes, is essential for viral replication and for establishing intracellular viral reservoirs that are protected from the immune system^10^,^11^,^12^.

Besides its well established role in the virus life cycle, increasing evidences suggest a role for exogenous p17 in deregulating the biological activity of different immune cells^13^,^14^,^15^,^16^, which are relevant in the context of viral pathogenesis. More recently, p17 was also found to exert chemokine and pro-angiogenic activities. These are mediated by p17 binding to CXCR1 and CXCR2, also termed IL-8RA and IL-8RB, and indeed, p17 was found to mimic some of the biological activities exerted by IL-8^17^,^18^,^19^.

Significant amounts of virion-free p17 are released from cells during active HIV-1 replication^1^. Moreover, p17 was found to be released in the course of combined antiretroviral therapy (cART) and during viral latency^16^,^20^. Several reports have shown that HIV-1 transcription can be efficiently induced by different stimuli^21^ even in the presence of protease inhibitors^22^, further suggesting the possibility that p17 synthesis and release may occur under cART. Moreover, latently infected resting CD4^+^ T cells were found to transcribe and translate *Gag* proteins without stimulation while in a latent state^23^, supporting the hypothesis that resting CD4^+^ T cells can synthesize HIV-1 *Gag* proteins without contributing to the spread of infection. All these findings strongly suggest that p17 may be chronically present in the infected microenvironment, even during pharmacological control of viral replication, thus in the absence of any HIV-1 protease activity.

Although p17 is devoid of signal sequence, the fact that nanomolar p17 concentrations are present in the plasma of HIV-1-infected patients^16^ indicates that this secretion process should be efficient. Nevertheless, if the production and release of p17 is conceivable during HIV-1 replication, the mechanism of p17 secretion during HIV-1 latency, in the absence of any viral protease activity, remains to be elucidated. Some cell proteins characterized by small dimensions, including numerous cytokines, are secreted despite their lack of signal sequence by unconventional export routes^24^,^25^. Previous studies showed that the highly basic region at the p17 NH_2_-terminus interacts with PI(4,5)P_2_^26^,^27^. PI(4,5)P_2_ is a phospholipid that is specifically concentrated within the inner leaflet of the plasma membrane wherein it recruits proteins involved in several important cell activities such as endocytosis, phagocytosis, exocytosis and cell adhesion^28^,^29^. Interestingly, the HIV-1 transactivating regulatory protein Tat has been recently found to be secreted in an unconventional manner after binding to PI(4,5)P_2_^30^. This prompted us to investigate possible unconventional routes of p17 secretion via PI(4,5)P_2_ interaction.

Herein we report that p17 is released in an active form by *Gag*-expressing cells. We show that secretion of p17 takes place at the plasma membrane and involves the interaction of p17 with PI(4,5)P_2_. The release of p17 occurs following its cleavage from Pr55^*Gag*^by cellular aspartyl proteases.

## Results

### A cellular ELISA to quantify p17 secretion by T cells

**Figure 1:**
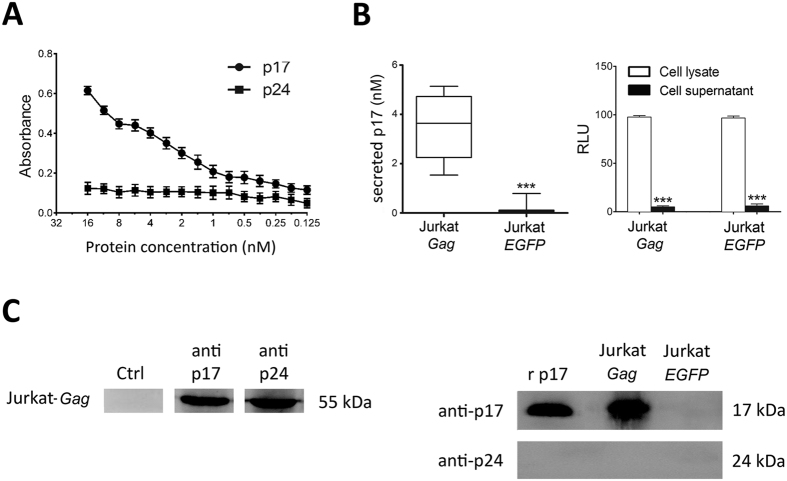
Quantitative p17 secretion assay. (**A**) Sensitivity of p17 antigen capture ELISA. To detect and quantify recombinant p17, the viral protein was resuspended in complete medium at different concentrations then added to anti-p17 mAb MBS15-coated ELISA microwells. Immunocomplexes were revealed using biotinylated mAb MBS34 followed by peroxidase-labelled streptavidin. Assay specificity was confirmed by the absence of p24 capture by mAb MBS15 when biotinylated anti-p24 mAb MBS-12 was used as a specific reagent in the revealing step. (**B**) Quantification of p17 released by Jurkat-*HIV-1*. Evaluation of p17 release in the cell culture supernatant was performed by cellular ELISA. Jurkat cells were nucleofected with or without the pNL4-3 expression vector. After 24 h, Jurkat-*HIV-1* were seeded onto ELISA plates pre-coated with anti-p17 mAb MBS15 and incubated further for 16 h to allow protein secretion and accumulation. Quantification of released p17 was performed using a standard curve generated with recombinant p17 as in (**A**). Amount of secreted p17 was calculated as mean ± SD of eight independent experiments performed in triplicate. The horizontal line in the middle of each box indicates the mean amount of secreted p17, whereas the top and bottom borders of the box mark the 75^th^ and 25^th^ percentiles, respectively (left panel). The release of p17 by Jurkat-*Gag* cells takes place in the absence of cell disruption as shown by the absence of CFSE in the cellular supernatant of Jurkat-*Gag* and Jurkat-EGFP. CFSE was quantified by fluorometric technique and 100% of RLU detection was calculated by performing a detergent-based cell disruption (right panel). Significance was assessed using Student’s *t* test; ***P < 0.001. (**C**) Detection of Pr55^*Gag*^ and p17 in cell extracts and cell culture supernatants. Jurkat-*Gag* cell extracts (left panel) and culture supernatants (right panel) were immunoprecipitated by a rabbit polyclonal antibody to p17 and then analyzed for p17 and Pr55^*Gag*^ expression by Western blot, using the anti-p17 mAb MBS-3 and the anti-p24 mAb MBS12 as specific reagents. Full-length blots were cropped for final display.

Our first aim was to set up a sensitive ELISA to quantify p17 in culture medium. A standard curve was generated using different concentrations of recombinant p17 diluted in complete medium. Capture of recombinant p17 was performed by the anti-p17 mAb MBS-15 coated on 96-well plates, whereas detection was obtained by adding biotin-conjugated anti-p17 mAb MBS-34 and peroxidase-labeled streptavidin. As shown in Fig. 1A, the limit of quantitation for the p17 capture ELISA was 0.125 nM of p17 since measurements of p17 detection at this concentration was the lowest with a p-value still significant (p < 0.001) as compared to the background signal. Specificity of the assay was demonstrated using HIV-1 p24 as unrelated protein in the ELISA, whose signal never significantly exceeded the level of background noise. Indeed, the HIV-1 capsid protein was not detected by the anti-p24 mAbs MBS-12^31^ even when it was added to the capture well at a concentration as high as 16 nM. Then, we developed a protocol for quantitating p17 secretion by living HIV-1-infected or by *Gag*-expressing cells. Jurkat cells, which are similar to most HIV-1 infected CD4^+^ T-cells^32^, were first labeled with CFSE (used as a control for cell lysis), then transiently nucleofected with pNL4-3 (Jurkat-*HIV-1*), with AG49CMV*Gag*-RTEm26CTE plasmid (Jurkat-*Gag*) or with *pEGFP*-N3 (Jurkat-*EGFP*; used as negative control and for evaluating nucleofection efficiency). Nucleofection efficiency was always higher than 80% as assessed by flow cytometic analysis. Twenty-four hours after nucleofection, 7 × 10^5^ Jurkat-*HIV-1*, Jurkat-*Gag* or Jurkat-*EGFP* cells were cultured for 24 h in complete medium in anti-p17 mAb MBS-15-coated wells of ELISA plates. Plate-bound p17, secreted by seeded Jurkat-HIV-1 or by Jurkat-*Gag* cells, was then quantified by ELISA. As previously reported^1^, p17 was easily detected in the supernatant of Jurkat-HIV-1 and the amount of secreted p17 ranged from 2.39 to 5.77 nM (mean ± SD:4.11 ± 0.75 nM). At the same time, p17 was also secreted by Jurkat-*Gag* cells and this occurred in the absence of any viral protease. The amount of p17 secreted by Jurkat-*Gag* ranged from 1.54 to 5.14 nM (mean ± SD: 3.47 ± 0.44 nM) (Fig. 1B, left panel). CFSE was not released in tissue culture supernatants of nucleofected cells, attesting to their viability during the whole period of cell culture (Fig. 1B, right panel).

In order to confirm the intracellular expression of Pr55^*Gag*^, as well as the release of extracellular p17 protein dissociated from Pr55^Gag^, we immunoprecipitated the two viral proteins from the cell lysate and from the supernatant of Jurkat-*Gag* cells, using a rabbit anti-p17 polyclonal antibody as specific reagent. As shown in Fig. 1C (left panel), Jurkat-*Gag* cell lysate showed the expression of Pr55^*Gag*^. On the other hand, when rabbit anti-p17 polyclonal antibody was used to precipitate p17 from the supernatant of Jurkat-*Gag* cells, p17 was the only protein detected in the immunoprecipitated sample (Fig. 1C, right panel). The proper molecular size of the viral protein suggests the presence of a specific cellular protease at work in cleaving p17 from Pr55^*Gag*^. At the same time, p24 was not detected in the immunoprecipitated material, thus excluding the presence of Pr55^*Gag*^ in the cell supernatant (Fig. 1C, right panel).

### Secreted p17 is biologically active

**Figure 2:**
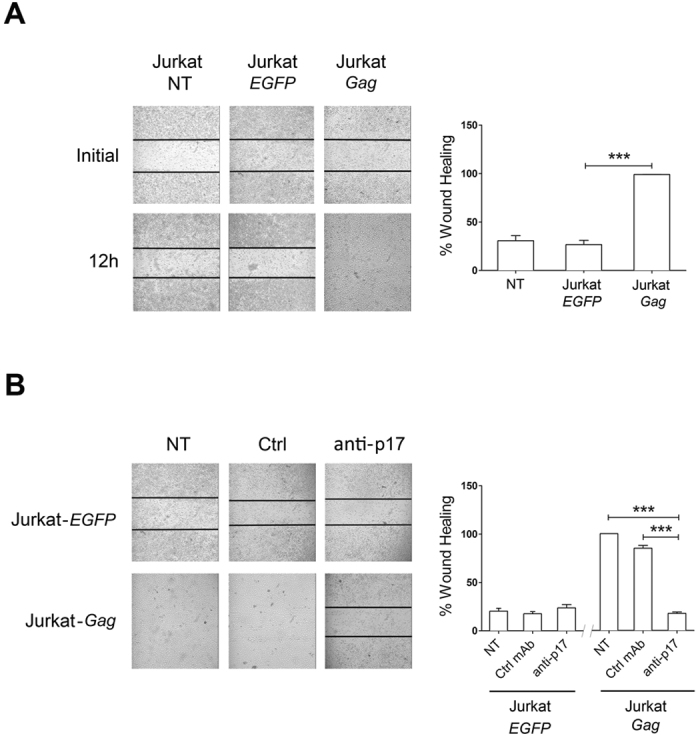
Jurkat-*Gag* cells release biologically active p17. (**A**) Ability of secreted p17 to stimulate endothelial cell migration. Jurkat, Jurkat-*EGFP* or Jurkat-*Gag* cells were added for 16 h to confluent HUVEC monolayers. After several washes with complete medium to remove added cells, HUVEC monolayers were scratched using a 200 μl pipette tip. Cell migration was recorded by light microscopy soon after (initial) and at 12 h after the wound. Pictures are representative of three independent experiments with similar results (magnification 10x). (**B**) Specificity of p17 biological activity. The same experiment as in (**A**) was performed in the presence of 1 μg/ml of neutralizing anti-p17 mAb MBS-3 or of an isotype matched control mAb (Ctrl). Neutralizing anti-p17 mAb MBS-3 (anti-p17) −but not an unrelated mAb (Ctrl)−blocked the pro-migratory activity of the p17 secreted by Jurkat-*Gag* cells. Images are representative of three independent experiments with similar results (magnification 10x). Graphs in the right panels represent quantitative analyses of wound-healing upon different co-culture conditions. Statistical analysis was performed by one-way ANOVA, and the Bonferroni’s post-test was used to compare data (***P < 0.001). NT indicates not treated cells.

To evaluate whether secreted p17 was biologically active, we took advantage of the ability of the protein to promote angiogenesis at a concentration as low as 0.58 nM^18^. In particular, we investigated the effect of p17 on human endothelial cell migratory activity using the wound healing assay^33^. This method highlights the ability of p17 to promote endothelial cell migration by sealing a confluent cell monolayer after a mechanical injury. HUVECs were grown on collagen-coated plates and starved for 24 h, then co-cultured with 1 × 10^7^ non-transfected Jurkat, Jurkat-*Gag* or Jurkat-*EGFP* cells. After an additional 24 h of incubation Jurkat cells were removed, confluent HUVEC monolayers were scratched with a 200-μl tip, then recorded over a period of 12 h. As shown in Fig. 2A, control HUVECs reached approximately 35% sealing (range from 28% to 44%) after 12 h of culture. The same level of sealing was reached when HUVECs were co-cultured in the presence of Jurkat-*EGFP*. On the other hand, HUVECs co-cultured with Jurkat-*Gag* cells reached 100% sealing, showing a considerable improvement in scrape wound repair ability. In order to confirm the direct involvement of p17 in promoting HUVEC migration, cell monolayers were cultured with Jurkat-*Gag* cells in the presence or absence of the p17 neutralizing mAb MBS-3 (2.5 μg/ml). Again, HUVECs reached 100% of wound sealing after 12 h of culture in p17-containing medium, even in the presence of an isotype-matched mAb. At the same time, the anti-p17 neutralizing mAb MBS-3 was strongly inhibitory (range from 77.2% to 88.8%) (Fig. 2B), thus confirming that enhancement of HUVEC migratory activity has to be ascribed to Jurkat-*Gag* secreted p17.

### Pr55^*Gag*^ binding to PI(4,5)P_2_ is required for p17 secretion

**Figure 3:**
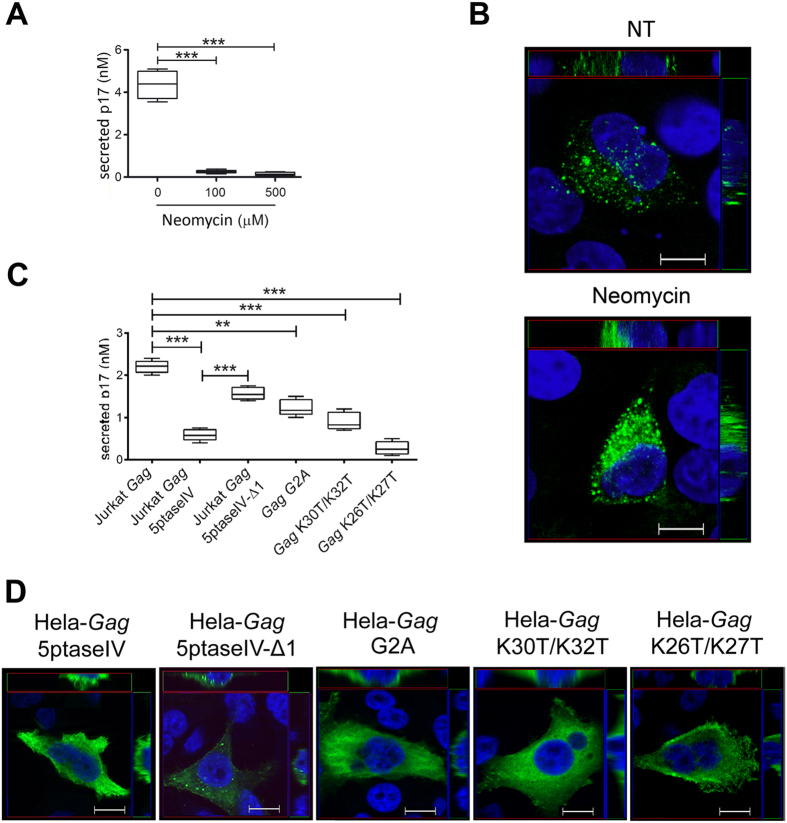
PI/4,5)P_2_ is involved in p17 secretion. (**A**) Quantification of extracellular p17 released by neomycin-treated Jurkat-*Gag* cells. Jurkat cells were transfected with AG49CMV*Gag*-RTEm26CTE and cultured for 24 h in the absence or in the presence of neomycin, at the concentration of 100 μM and 500 μM. After washing, cells were added to anti-p17 mAb MBS15-coated wells for 16 h at 37 °C in the absence or in the presence of 100 μM or 500 μM of neomycin. Plate-bound p17, secreted by seeded Jurkat-*Gag* cells, was then quantified by ELISA. Amount of secreted p17 was calculated as mean ± SD of three independent experiments performed in triplicate. Statistical analysis was performed by one-way ANOVA, and the Bonferroni’s post-test was used to compare data (***P < 0.001). (**B**) Intracellular localization of p17 in *Gag*-expressing cells. *Gag*-expressing HeLa (HeLa-*Gag*) cells, soon after nucleofection with AG49CMV*Gag*-RTEm26CTE, were cultured for 16 h at 37 °C in the absence (NT) or in the presence of neomycin (100 μM). Cells were then fixed and permeabilized as described in the materials and methods section. Cells were then stained with biotinylated anti-p17 mAb MBS-3 followed by Alexa488-streptavidin and 4′,6-diamidino-2-phenylindole. Analysis was performed by confocal fluorescence microscopy. Images display mAb MBS-3 signals in green and cell nuclei in blue. z-Stack sections and orthogonal z reconstitution are also shown. Scale bar, 10 μM. (**C**) Quantification of p17 released by Jurkat cells co-nucleofected with AG49CMV*Gag*-RTEm26CTE and 5ptaseIV or 5ptaseIV-Δ1 mutant expression plasmids, or by Jurkat cells expressing G2A, K30T/K32T or K26T/K27T *Gag* mutants. Amount of secreted p17 was calculated as mean ± SD of three independent experiments performed in Triplicate. Statistical analysis was preformed by one-way ANOVA, and the Bonferroni’s post-test was used to compare data (**P < 0.01, ***P < 0.001). (**D**) Intracellular localization of p17 in HeLa cells co-expressing *Gag* and 5ptaseIV or 5ptaseIV-Δ1 and in HeLa cells expressing G2A, K30T/K32T or K26T/K27T *Gag* mutants. Cells were stained with biotinylated anti-p17 mAb MBS-3 followed by Alexa488-streptavidin (green) and 4′,6-diamidino-2-phenylindole (in blue). Representative median confocal sections of at least 20 cells per conditions are shown. z-Stack sections and orthogonal z reconstitution are also shown. Scale bar, 10 μM.

PI(4,5)P_2_ is a phospholipid that is specifically concentrated within the inner leaflet of the plasma membrane^28^,^29^. Several groups have examined the interaction between p17 and PI(4,5)P_2_, and their studies revealed that the highly basic residues (HBR) in the p17 NH_2_-terminal region are responsible for such interaction^26^,^34^,^35^,^36^,^37^. Therefore, it is likely that precursor Pr55^*Gag*^processing has to occur after p17 binding to PI(4,5)P_2_. To assess the role of p17/PI(4,5)P_2_ interaction in the process of p17 secretion, we evaluated the presence of viral protein in the supernatant of Jurkat-*Gag* treated with neomycin, a polycationic aminoglycoside antibiotic which binds to PI(4,5)P_2_ impairing the binding of PI(4,5)P_2_ ligands by steric hindrance^38^. Neomycin, in concurrence with previous results^30^, was deprived of any cell toxicity at concentrations ranging from 100 to 500 μM. At both concentrations, neomycin was found to completely inhibit p17 secretion (Fig. 3A). Evaluation of the intracellular localization of p17 in the presence of neomycin was then carried out by confocal microscopy. HeLa cells were nucleofected with AG49CMV*Gag*-RTEm26CTE (HeLa-*Gag*), then stained with the anti-p17 mAb MBS-3. Representative confocal images of untreated cells showed p17 as a punctate staining at the plasma membrane, whereas cells treated with neomycin displayed a diffuse staining in the cytoplasm (Fig. 3B). These results confirm that displacement of Pr55^*Gag*^/PI(4,5)P_2_ interaction by neomycin prevents its localization to the plasma membrane and p17 secretion. To further confirm a key role of PI(4,5)P_2_ in the p17 secretion mechanism, we reduced cellular PI(4,5)P_2_ levels by overexpressing 5ptaseIV, an enzyme that depletes cellular PI(4,5)P_2_^39^, and examined the impact of PI(4,5)P_2_ depletion on p17 secretion. As shown in Fig. 3C, p17 secretion was strongly impaired in Jurkat cells co-nucleofected with AG49CMV*Gag*-RTEm26CTE and the 5ptaseIV expression vector (Jurkat-*Gag-5ptaseIV*). At the same time, Jurkat cells co-nucleofected with AG49CMV*Gag*-RTEm26CTE and a vector expressing the mutant form of 5ptaseIV (Jurkat-*Gag-5ptaseIV-Δ1*) lacking the 5-phosphatase signature domain, released p17 at concentrations not significantly different from those detected in the supernatant of Jurkat-*Gag* cells. We next examined whether localization of *Gag* to the plasma membrane was affected by PI(4,5)P_2_ depletion. HeLa cells were co-nucleofected with AG49CMV*Gag*-RTEm26CTE and the 5ptaseIV expression vector (HeLa-*Gag-5ptaseIV*) and analysed by confocal microscopy. As shown in Fig. 3D, HeLa-*Gag* overexpressing 5ptaseIV displayed a diffuse distribution of the viral protein in the cytoplasm. In contrast, HeLa-*Gag* overexpressing the mutant form of 5ptseIV (HeLa-*Gag-5ptaseIV-Δ1*) showed a punctate staining at the plasma membrane resembling that observed in HeLa-*Gag*.

Different authors have demonstrated that *Gag* binding to PI(4,5)P_2_ triggers exposure of the amino-terminal myristic acid moiety that inserts into the membrane, anchoring and stabilizing *Gag*-membrane interaction^7^,^40^,^41^. Therefore, we asked whether elimination of the myristic acid moiety within p17 was impairing its secretion. As shown in Fig. 3C, cells expressing a not myristoylated G2A mutated *Gag (Gag*G2A) were impaired in their capability to secrete p17. At the same time, *Gag*G2A-expressing cells showed a lower p17 signal at the cell surface compared to cells expressing *Gag*, but instead typically displayed a greater accumulation of p17 throughout the cytoplasm.

To further support the hypothesis that p17 secretion relies on anchorage of Pr55^*Gag*^ to plasma membrane through PI(4,5)P_2_ interaction, we evaluated the role of p17 HBR in p17 secretion by using different mutated Pr55^*Gag*^ polyproteins. We first generated a *Gag* mutant with reduced PI(4,5)P_2_ binding, based on previous reports showing that mutation of lysine 30 and 32 to threonine (K30T/K32T) within the p17 HBR impairs *Gag*/PI(4,5)P_2_ interaction and relocalizes *Gag* from the plasma membrane to intracellular compartments^10^. As shown in Fig. 3C, secretion of p17 by Jurkat cells expressing the *Gag*K30T/K32T was significantly reduced − but not completely abolished − compared to cells expressing *Gag*. Moreover, as expected, confocal images of cells expressing the *Gag*K30T/K32T showed a redistribution of p17 in the cytoplasm (Fig. 3D), as compared to the plasma membrane-associated expression in cells nucleofected with *Gag* (Fig. 3B). At the same time, a *Gag*K26T/K27T mutant with elevated PI(4,5)P_2_-independent binding to plasma membrane^36^ showed a dramatic impairment of p17 secretion compared to *Gag* (Fig. 3C) and a marked p17 intracellular accumulation (Fig. 3D). This result indicates that promiscuous *Gag* binding to membrane is not sufficient to promote p17 cleavage and secretion and highlights the role of specific amino acid residues within the p17 HBR in ensuring specific *Gag*/PI(4,5)P_2_ interaction, thus allowing p17 proteolytic cleavage from Pr55^*Gag*^ and its release in the extracellular microenvironment. Altogether, our data show that targeting *Gag* to the plasma membrane through PI(4,5)P_2_ interaction is the only feasible mechanism for p17 secretion.

### Molecular basis for p17 secretion

**Figure 4:**
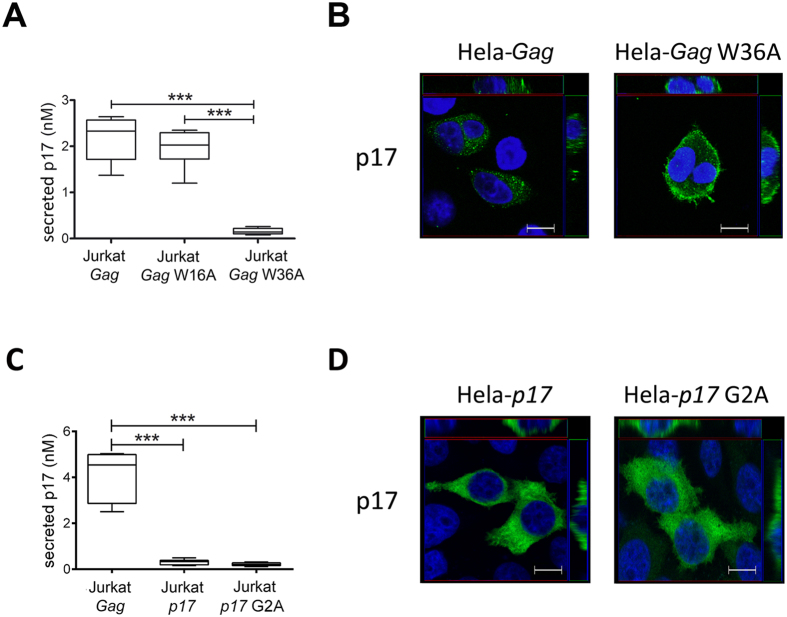
Secretion of p17 does not occur if its W36 residue is mutated or when it is expressed alone. (**A**) Quantification of extracellular p17 released by Jurkat cells expressing W16A or W36A *Gag* mutants. Cells were nucleofected with construct expressing wild type *Gag* or *Gag* mutants containing W16A and W36A amino acid changes. After 24 h later cells were cultured for 16 h onto ELISA plates and secreted p17 was quantified by cellular ELISA. Box plots represent statistical analyses of five independent experiments. Significance was assessed using one-way ANOVA and the Bonferroni’s post-test was used to compare data; ***P < 0.001. (**B**) Confocal analysis of *Gag* localization. HeLa nucleofected with the same constructs as in (**A**) were stained with biotinylated anti-p17 mAb MBS-3 followed by Alexa488-streptavidin (green) and 4′,6-diamidino-2-phenylindole (in blue). Representative median confocal sections of at least 20 cells per conditions are shown. Images highlight that W36A mutation leads to protein accumulation in proximity of the plasma membrane. z-Stack sections and orthogonal z reconstitution are also shown. Scale bar, 10 μM. (**C**) Quantification of extracellular p17 released by Jurkat cells expressing wild type p17 (myr^+^) or its G2A mutant (myr^−^). Cells were nucleofected with the construct that expresses wild type *Gag*, or with constructs that express *p17* or a mutant containing the G2A amino acid change (*p17* G2A). Cells were recovered 24 h later, then cultured for 16 h onto ELISA plates and secreted p17 was quantified by cellular ELISA as in Fig. 1. Box plots represent statistical analyses of five independent experiments. Significance was assessed using one-way ANOVA and the Bonferroni’s post-test was used to compare data; ***P < 0.001. (**D**) Confocal analysis of p17 localization. HeLa nucleofected with the same constructs as in (**C**) were stained with biotinylated anti-p17 mAb MBS-3 followed by Alexa488-streptavidin (green) and 4′,6-diamidino-2-phenylindole (in blue). Representative median confocal sections of at least 20 cells per conditions are shown. Images highlight that p17 alone is trapped in the cell cytosolic compartment regardless of whether or not it is myristoylated. z-Stack sections and orthogonal z reconstitution are also shown. Scale bar, 10 μM.

The structure of the p17/PI(4,5)P_2_ complex has been recently defined^26^. NMR data are consistent with a single binding mode which involves, in addition to the p17 highly basic domain, the participation of other parts of the molecule. Among hydrophobic residues that could be involved in PI(4,5)P_2_ interaction, p17 contains a well-preserved tryptophan at position 36 (W36). W36 was found to be important in interacting with both the glycerol moiety and the phosphoinositide head group of PI(4,5)P_2_ and in participating to the p17/PI(4,5)P_2_ complex stabilization^26^. Another tryptophan at position 16 (W16) is also located near the p17 highly basic domain. Binding of p17 to p17/PI(4,5)P_2_ results in small local conformational changes that reposition hydrophobic residues such as W16^26^. To examine whether W16 and/or W36 were important in participating to the mechanism of p17 secretion, we determined the presence of p17 in the supernatant of Jurkat cells nucleofected with AG49CMV*Gag*W16A-RTEm26CTE (Jurkat-*Gag*W16A) or AG49CMV*Gag*W36A-RTEm26CTE (Jurkat-*Gag*W36A) constructs carrying respectively a W16Alanin (A) and a W36A mutation in the *Gag* sequence. As shown in Fig. 4A, W36A mutation strongly impaired p17 secretion, as assessed by the low amount of extracellular p17 detected in Jurkat-*Gag*W36A supernatants (0.08–0.26 nM). On the other hand, Jurkat-*Gag*W16A secreted approximately the same amount of p17 (1.2–2.35 nM) as those nucleofected with AG49CMV*Gag*-RTEm26CTE (1.37–2.64 nM). This finding indicates the importance of W36 − but not of W16 − in the p17 secretion mechanism. Confocal analysis performed on nucleofected HeLa cells revealed that differently to HeLa-*Gag*, HeLa cells expressing *Gag*W36A displayed a uniform distribution of the viral protein in the cytoplasm and at near all areas of the plasma membrane (Fig. 4B). This evidence suggests that mutation in W36 does not affect the ability of p17 to reach the plasma membrane.

In order to assess if p17 needs to be part of Pr55^*Gag*^ to reach plasma membrane and be subsequently cleaved and secreted, or if p17 can be also secreted after Pr55^Gag^ enzymatic digestion in intracellular compartments other than plasma membrane, further experiments were conducted by expressing p17 alone in cells. There is considerable evidence that PI(4,5)P_2_ binds to a conserved cleft on p17 and promotes myristate exposure and tight membrane binding^26^. Therefore, we expressed myristoylated (myr^+^) and not myristoylated (myr^−^) p17 in Jurkat cells by nucleofecting them with AG49CMV*p17*-RTEm26CTE (Jurkat-*p17*) or AG49CMV*p17*G2A-RTEm26CTE (Jurkat-*p17*G2A) constructs, carrying respectively a wild type and a G2A mutation in the *p17* sequence and checked for secretion of p17 in the supernatant. As shown in Fig. 4C, we found that both myr^+^ and myr^−^ p17s are not secreted although, using confocal microscopy, they are well expressed in the cell cytosolic compartment (Fig. 4D). This result is in agreement with previous data showing that within full-length Pr55^*Gag*^, the membrane targeting signal is exposed and can direct Pr55^*Gag*^ to the plasma membrane, whereas in the context of p17 alone, this signal is hidden and unable to confer plasma membrane binding^40^,^41^. This data suggests that p17 secretion occurs only after Pr55^Gag^ targeting to PI(4,5)P_2_ on the plasma membrane.

### Secretion of p17 is modulated by inhibitors of cellular aspartyl proteases

**Figure 5:**
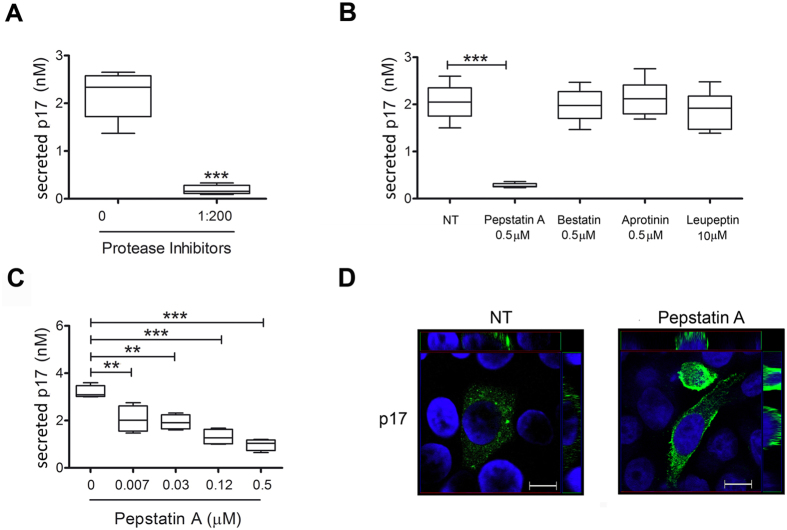
Secretion of p17 protein depends upon cleavage by cellular proteases. (**A**) Evaluation of p17 secretion in the presence of inhibitors of different cellular proteases. Jurkat-*Gag* were cultured for 16 h in ELISA plates in the presence or absence of a cocktail containing inhibitors of different cellular proteases. Secreted p17 was quantified by cellular ELISA as in Fig. 1. Box plots represent statistical analyses of six independent experiments. Significance was assessed using Student’s t test (***P < 0.001). (**B**) The same experiments as in (**A**) were performed using inhibitors of specific cellular proteases as Pepstatin A, Bestatin, Aprotinin and Leupeptin or (**C**) different doses of the aspartyl protease inhibitor Pepstatin A. Box plots represent statistical analyses of six independent experiments. Significance was assessed using one-way ANOVA and the Bonferroni’s post-test was used to compare data; **P < 0.01, ***P < 0.001. (**D**) Confocal analysis of p17 localization in Pepstatin-A-treated cells (0.5 μM) shows that p17 accumulates at the plasma membrane. z-Stack sections and orthogonal z reconstitution are also shown. Scale bar, 10 μM.

Cellular ELISA and Western blot confirmed the presence of properly sized and biologically active p17 in the supernatant of Jurkat-*Gag* cells, suggesting that *Gag* polyproteins are cleaved by cellular proteases. To confirm this hypothesis, a cocktail of different cellular protease inhibitors were added to Jurkat-*Gag* cells into the ELISA plate. Data obtained after 16 h of culture showed that the cocktail of inhibitors completely blocked p17 secretion (Fig. 5A). Therefore, we examined in detail which inhibitor(s) of the cocktail was responsible for this effect. Different amounts of each protease inhibitor included in the original cocktail were used according to the higher dose without any toxic effect on Jurkat-*Gag*. In particular, Pepstatin-A (an inhibitor of aspartyl proteases), Aprotinin (a serine protease inhibitor), and Bestatin (an aminopeptidase inhibitor) were used at 0.5 μM, whereas Leupeptin (an inhibitor of serine and cysteine protease) was used at the concentration of 10 μM. As shown in Fig. 5B, Pepstatin A almost completely abolished p17 secretion, whereas Bestatin, Aprotinin and Leupeptin did not show any significant effect in modulating p17 secretion. The capability of Pepstatin A to inhibit p17 secretion was found to be statistically significant even at a concentration as low as 0.007 μM (Fig. 5C). As a result of Pepstatin A activity, p17 expression was scattered within the cell cytoplasm but it resulted to be more pronounced at the plasma membrane (Fig. 5D). This result suggests that Pepstatin A favors p17 accumulation in *Gag-*expressing cells by interfering with its enzymatic cleavage from Pr55^*Gag*^.

### Renin, Cathepsin-D and γ-secretase cleave p17 from the *Gag* polyprotein

**Figure 6:**
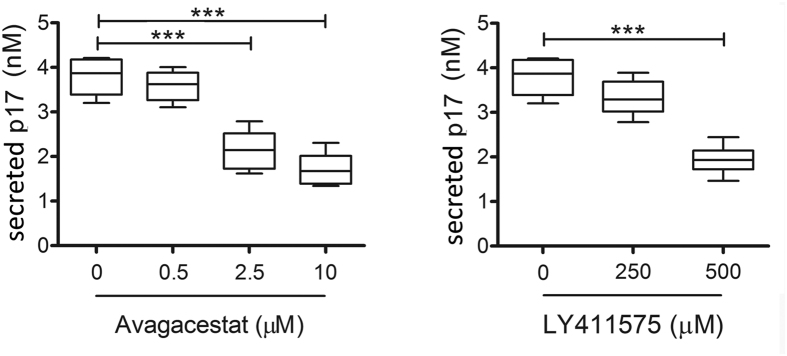
Secretion of p17 protein is blocked by specific inhibitors of γ-secretase inhibitors. Jurkat-*Gag* were cultured for 16 h in ELISA plates in the presence or absence of the specific γ-secretase inhibitors Avagacestat and LY411575. Box plots represent statistical analyses of six independent experiments. (**A**) Avagacestat and (**B**) LY411575 inhibit p17 release in a dose-dependent manner. Significance was assessed using one-way ANOVA and the Bonferroni’s post-test (***P < 0.001).

**Figure 7:**
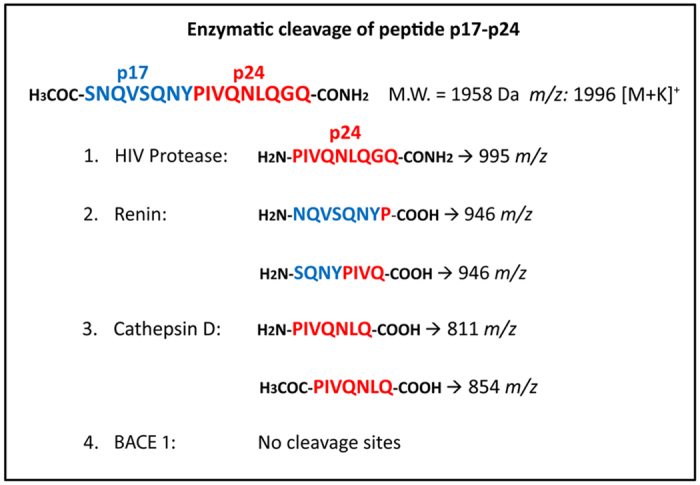
Summary of cleavage patterns of peptide p17-p24 by HIV protease, Renin, Cathepsin D and BACE 1. The residues formed following incubation with each enzyme are reported.

Pepstatin A is an highly selective inhibitor of aspartyl proteases, forming a complex with γ-secretase^42^, Renin and Cathepsin-D^43^,^44^, with the exception of an aspartyl protease with β-secretase activity named BACE-1^45^. In order to evaluate the capability of γ-secretase to cleave p17 from the precursor Pr55^*Gag*^, we examined the effect of two specific inhibitors of γ-secretase, namely Avagacestat and LY411575, on p17 secretion. As shown in Fig. 6, Avagacestat and LY411575 were both able to interfere with p17 secretion from Jurkat-*Gag* cells. However, both inhibitors only partially impaired protein secretion, being able to reduce p17 detection of approximately 53.9% and 46.2% using Avagacestat and LY411575 at the not toxic concentration of 10 μM and 500 μM, respectively. Following the only partial decrease of p17 secretion observed by blocking γ-secretase activity, we asked whether other aspartyl proteases as Renin, Cathepsin-D or BACE-1 might be involved in the p17 cleavage from the *Gag* polyprotein. To this purpose, the enzymatic activity of these proteases was tested *in vitro* on a synthetic peptide representative of the precursor Pr55^*Gag*^ region containing the cleavage site of p17 from p24 (p17/p24). As summarized in Fig. 7, HIV-1 protease cleaved specifically the p17-p24 peptide only between the tyrosine (Y) 132 of p17 and proline (P) 1 of p24 (see also Fig. S1). Interestingly, both Renin and Cathepsin-D were found to hydrolyze the p17-p24 peptide. Renin operated a complex proteolysis, by generating two different peptides: the first one resulted from the cleavage between asparagine (N) 126 of p17 and P1 of p24 and the second one between serine (S) 129 of p17 and glutamine (Q) 4 of p24 (see also Fig. S2). Therefore, Renin is likely to produce a 4 amino acid shorter p17 (p17Δ4) and a protein containing an additional amino acid (P) at its C-terminus belonging to the p24 molecule. Also Cathepsin-D was found to cleave the peptide in two sites, the first one is the canonical cleavage site for HIV-1 protease, located between Y132 of p17 and P1 of p24 and the second one between Q7 and glycine (G) 8 of p24. This finding attests that Cathepsin-D proteolysis of the *Gag* polyprotein is likely to result in the production of a full-lenght p17 and of a p24 devoid of seven aminoacids at its N-terminus (see also Fig. S3). Our results show that the consensus primary sequence, at least for Renin, does not overlap with that of the HIV-1 protease. This finding is in agreement with previous observation showing that there is no consensus sequence for proteolysis among different substrates for aspartyl proteases^46^. Interestingly, BACE-1 was totally unable to hydrolyze the peptide (see also Fig. S4). These results show that Cathepsin-D, Renin and γ-secretase are likely participating to p17 cleavage from the *Gag* polyprotein. The possibility that cellular aspartyl proteases may have more cleavage sites on p24 cannot be ruled out. Further studies using p24 peptides or the entire *Gag* polyprotein as specific substrates for cellular aspartyl proteases are necessary to test this hypothesis.

### Impact of the C-terminus on p17 structure and bioactivity

**Figure 8:**
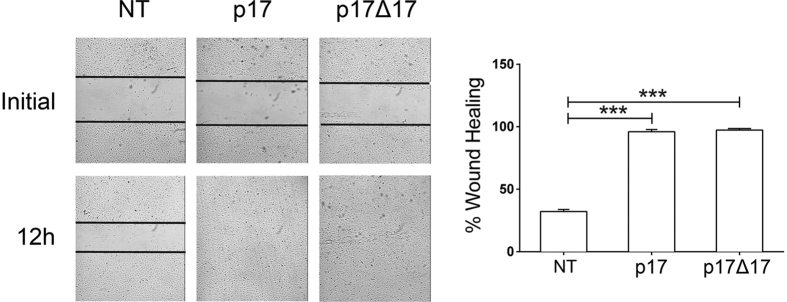
A C-terminally truncated p17 protein with 17 disordered C-terminal residues excised (p17Δ17) is biologically active. Ability of p17Δ17 to promote endothelial cell migration. Confluent HUVEC monolayers were scratched using a 200 μl pipette tip and cultured for 12 h at 37 °C with medium alone (NT) or containing 10 ng/ml of p17 (used as a positive control) or p17Δ17. Cell migration was recorded by light microscopy soon after (initial) and at 12 h after the wound. Images are representative of three independent experiments with similar results (magnification 10x). Graphs in the right panels represent quantitative analyses of wound-healing upon different culture conditions. Statistical analysis was performed by one-way ANOVA, and the Bonferroni’s post-test was used to compare data (***P < 0.001). NT indicates not treated cells.

Proteolysis of the *Gag* polyprotein operated by cellular aspartyl proteases is likely to produce p17s slightly different in their C-terminus in terms of length. Since we found that secreted p17 is biologically active, further experiments were conducted to understand the role of the C-terminus in the p17 action. Recent data point to the C-terminus of p17 as partially α-helical (H5 helix) ending in a disordered region which would have little impact on protein folding and stability. The disordered C-terminal region of p17 varies in length in all known solution and crystal structures, where the last H5 α-helix terminates at residues 109–122 depending on experimental conditions^47^,^48^. We synthesized a C-terminally truncated p17 protein with 17 disordered C-terminal residues excised (p17Δ17) on the basis of the crystal structure of p17^48^. As predicted, NH-NH NOEs of synthetic p17Δ17 (red) nicely overlapped with those of the entire p17 molecule (black), suggesting that p17Δ17 adopted an α-helical conformation highly similar, if not identical, to p17 (Fig. S5). Notably, we found that both synthetic p17 and p17Δ17 were functionally identical in sustaining HUVEC motility in a wound healing assay (Fig. 8), fully corroborating the structural findings by NMR spectroscopy. This finding demonstrates that the disordered region in the C-terminus of p17 is irrelevant for the viral protein’s biological activity.

## Discussion

Several evidences, collected in the past by different investigators, were already highly suggestive of cellular mechanisms at work to operate p17 secretion. Immunofluorescence staining of HIV-1-infected cell lines showed that different antigenic regions of p17 were found to be randomly expressed on the outer leaflet of live cell plasma membrane during HIV-1 replication^49^,^50^. In particular, at least three regions of p17 (aa.12–29, 53–87, 87–115) were constantly detected on the cell surface by different anti-p17 mAbs, used as specific reagents, whereas other epitopes, even conformational, were occasionally expressed. Based on the evidence that these mAbs lacked HIV-1 neutralizing activity, the authors concluded that the reactive epitopes of p17 were not the portions of viral protein in the virion itself, but more likely short polypeptide chains of p17 transported to the surface of HIV-1-infected cells^50^. More interestingly, p17 secretion has been hypothesized also in patients undergoing successful cART, since the viral protein was easily detected at nM concentration in the blood of cART-treated HIV-1-seropositive patients^16^. Moreover, p17 was detected in abundant amounts in the lymph node germinal centers^20^ and in the liver of aviremic cART-treated patients^18^.

**Figure 9:**
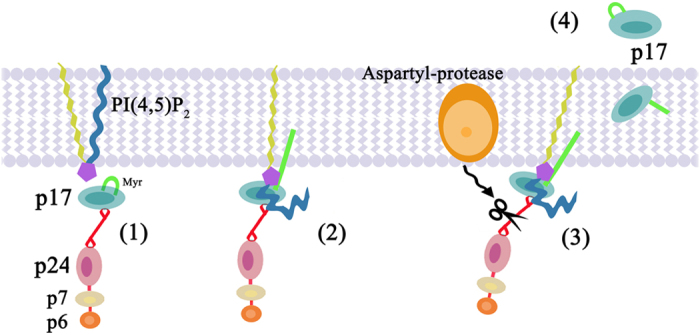
Hypothetical model of p17 secretion from *Gag*-expressing cells. The cytosolic Pr55^*Gag*^, a polyprotein composed of the matrix protein p17 − whose NH_2_-terminal myristic acid moiety in its sequestered conformation is in light green −, the capsid protein p24, the nucleocapsid protein p7, and the p6 domains (**1**), is recruited to the cellular membrane by PI(4,5)P_2_, according to the model proposed by Saad *et al*.^26^ (**2**). PI(4,5)P_2_ association with the p17 highly basic domain induces a conformational change enabling the membrane-embedded aspartyl-protease to cleave p17 from the polyprotein Pr55^*Gag*^ (**3**). As a Pr55^*Gag*^-free protein, p17 then moves to the extracellular space through an unconventional secretion mechanism (**4**).

Although hypothesized, the process of p17 secretion has remained poorly characterized due to difficulty in discriminating whether the presence of viral protein in the extracellular microenvironment was resulting from a mechanism of active cellular secretion or a product of viral particle degradation during active HIV-1 replication^1^. Meanwhile, several studies showed that extracellular p17 was indeed a viral toxin that could affect the biological activity of different cell types^17^,^18^,^51^. A recent study^23^ shed new light on the possibility that even during HIV-1 latency, cells can produce *Gag* polyproteins without supporting virus assembly and therefore, spreading infection. According to the authors, this was due to the presence of sufficient unspliced RNA to result in nuclear export and translation of *Gag*, whereas the same could not be said for *Env*. This finding is in agreement with previous *in vivo* studies showing that a population of resting cells can transcribe and translate HIV-1 and SIV proteins^21^,^22^. These findings prompted us to investigate whether *Gag* could be processed in the absence of HIV-1 protease and give rise to p17 proteins capable of being released, in a biologically active form, in the extracellular microenvironment. Evidence that cellular proteases may process the *Gag* polyprotein has been recently provided by Chandel *et al*.^52^ who showed that Renin is able to cleave *Gag* polyproteins to p24. This is not surprising since Renin and HIV-1 protease are both aspartyl proteases and similarly structured, so much so that HIV-1 protease inhibitors have been generally constructed on Renin structure^53^. As summarized in Fig. 9, here we provide evidence, for the first time, that the matrix protein p17 is digested from the *Gag* polyprotein by cellular intra-membrane aspartyl proteases following its binding to PI(4,5)P_2_. Then, although p17 is devoid of signal sequence, it is secreted in a biologically active form by cells using an unconventional secretion pathway. These findings offer to explain why *Gag* proteins can be released also during HIV-1 latency, when the viral protease is not synthesized^23^,^52^.

Data obtained show that Pr55^*Gag*^ binding to PI(4,5)P_2_ on plasma membrane is an essential step in promoting p17 cleavage and secretion. In fact, reducing cellular PI(4,5)P_2_ levels by overexpressing 5ptaseIV^40^, strongly impaired p17 secretion. Beyond the role of specific amino acids included in the HBR in p17/PI(4,5)P_2_ interaction, our data highlight also the importance of specific amino acid residues within HBR in promoting and/or stabilizing such interaction and consequently, in allowing p17 secretion. In particular, lowering Pr55^Gag^/PI(4,5)P_2_ interaction by mutating lysine 30 and 32 to threonine^35^ or, even more, by mutating lysine 26 and 27 to threonine^36^, strongly affected p17 secretion.

Unexpectedly, here we show that p17 secretion requires W36 in the p17 primary sequence to occur. This finding can be, at least in part, explained by the knowledge that the side chain of W36 interacts with the glycerol moiety and with the C_4_-acyl chain of PI(4,5)P_2_^26^. It is worth noting that W36 is a highly conserved amino acid and possibly evolutionarily relevant, given that it is maintained in its ancestor SIV matrix protein, whose extracellular biological activities have been recently found to resemble, for many aspects, those of the HIV-1 p17^54^. Such high conservation, that occurs for all p17 amino acid residues strictly involved in PI(4,5)P_2_ interaction, may be necessary for sites that interact with cellular constituents that do not undergo evolutionary changes on the timescale of HIV-1 replication. The role of the p17 HBR in key events in the viral life cycle is well known^1^. Indeed, virions carrying mutations in the p17 HBR were found to harbor not only an early entry defect, but also to display a severe impairment in their ability to incorporate envelope and participate in viral assembly^55^,^56^. In this respect, and despite the critical role in sustaining p17 export, it will be interesting to examine the impact of W36 mutation in the HIV-1 life cycle and in particular, in assembly and release of infectious HIV-1 particles.

Secreted p17 was found to have a proper molecular size following immunoprecipitation and to be biologically active. However, enzymatic cleavage of p17 from the *Gag* polyprotein exerted by Renin is likely to generate p17s characterized by slightly different lengths in their C-terminus, compared to the p17 cleaved by the HIV-1 protease and Cathepsin D. This finding called for further studies elucidating the role of p17 C-terminus in the viral protein biological activity. Data here presented show that the C-terminus of p17 is devoid of any biological activity and that deletion of as much as 17 amino acids in this region does not affect folding and biological activity of the viral protein. This finding is in agreement with several observations indicating that the functional region of p17 is located in a partially unfolded α-helix at the protein NH_2_-terminal region^13^. Moreover, the p17 C-terminus is highly flexible and with high level of predicted intrinsic disorders, which subtends to lack of function for protein activity. Indeed, mutations in the p17 C-terminus generate proteins that do not affect viral replication and infectivity^53^ and do not compromise p17 binding to its cellular receptors.

In conclusion, the function of extracellular p17 in HIV-1 infection and pathogenesis might be more significant than previously thought. In fact, our data sustain the possible role of extracellular p17 in HIV-1-related diseases which detrimentally affect successfully cART-treated patients. It is worth noting that recent data have already highlighted the possible contribution of extracellular p17 to support aberrant angiogenesis and promote vascular diseases^19^. Moreover, it is now becoming evident that p17 and its variants, may play a role in B-cell lymphoma growth and dissemination^19^,^57^,^58^. At present, the p17 protein is the target of a therapeutic vaccine aimed at boosting and better directing the immune response against the functional epitope involved in the p17/p17 receptors interactions^59^. At the same time, inhibitors of the p17/PI(4,5)P_2_ interaction^60^ as well as inhibitors of γ-secretase^61^ are major targets for drug development. Identification of the fine mechanisms underlying p17 secretion will help to develop further therapeutic interventions for eliminating the presence of the viral toxin in the extracellular microenvironment.

## Methods

### Expression vectors

pNL4-3 vector, containing a full infectious provirus, HIV-1_NL4-3_ was a kind gift of Dr. Anna Cereseto (University of Trento, Italy). Tat-independent AG49CMVIL15-RTEm26CTE vector was kindly provided by Dr. Barbara Felber (NCI, Frederick). The *Gag*-expressing plasmid AG49CMV*Gag*-RTEm26CTE was generated by replacing the *IL-15* gene within the *Sal*I/*Eco*RI restriction sites of AG49CMVIL15-RTEm26CTE with the *Gag* gene derived from the pNL*Gag*RTEm26-CTE vector, kindly provided by Dr. Barbara Felber (NCI, Frederick). Similarly, a myristoylated (myr^+^) and not myristoylated (myr^−^) p17-expressing plasmid AG49CMV*p17*-RTEm26CTE was generated by replacing the Gag gene within the *Sal*I/*Eco*RI restriction sites of AG49CMV*Gag*-RTEm26CTE with the p17 gene. *Gag* mutants constructs were generated from AG49CMV*Gag*-RTEm26CTE using the Quickchange site-directed mutagenesis kit (Stratagene, La Jolla, CA). Mutagenesis resulted in a tryptophan 16 or 36 to alanine (W16A or W36A, respectively), in a glycine 2 to alanine (G2A), in a lysine 30 and 32 to threonine (K30T/K32T) and in a lysine 26 and 27 to threonine (K26T/K27T). The *pEGFP*-N3 commercial plasmid (BD Biosciences, San Diego, CA) was used in some experiments as control vector. The 5ptaseIV expression plasmid, pcDNA4TO/Myc5ptaseIV, was a gift from P. Majerus (Washington University School of Medicine, St. Louis). The 5ptaseIV mutant lacking the phosphatase signature domain (Δ1) was a gift from A. Ono (University of Michigan medical School, Ann Arbor).

### Cells

HeLa and Jurkat cell lines were maintained in RPMI-1640 (Sigma, Milan, Italy) supplemented with 10% Fetal Bovine Serum (FBS) (Sigma) (complete medium). Human umbilical vein endothelial cells (HUVECs) have been previously developed and characterized^62^. Cells were cultured in Endothelial Cell Growth Medium (EGM™, Lonza, Milan, Italy) supplemented with 10% FBS.

### Recombinant proteins and monoclonal antibodies to p17

HIV-1 matrix protein p17, HIV-1 capsid protein p24 and glutathione S-transferase (GST) were produced and purified as previously described^63^. The absence of endotoxin contamination (<0.25 endotoxin units/mL) in protein preparations was assessed by Limulus amoebocyte assay (Associates of Cape Cod, East Falmouth, MA). Monoclonal antibodies (mAbs) to p17 capable of neutralizing (MBS-3 and MBS-34) or not (MBS-15) p17/receptors interaction, and therefore all the p17 biological activities, were produced in our laboratory^13^,^64^. Mab MBS-34 was also biotinylated using AH-N-Hydroxysuccinimido-biotin reagent (AH–NHS–biotin; SPA, Milan, Italy) according to the manufacturer’s instructions.

### p17 secretion Assay

Jurkat cells were nucleofected with pNL4-3, AG49CMV*Gag*-RTEm26CTE, AG49CMV*Gag*W16A-RTEm26CTE, AG49CMV*Gag*W36A-RTEm26CTE, AG49CMV*p17*-RTEm26CTE, and AG49CMV*p17*G2A-RTEm26CTE using the Amaxa Nucleofector II device (Lonza, Amboise, France) and the Ingenio Electroporation Universal kit (Mirus, Madison, USA). Twenty-four hours after nucleofection, cells were washed with RPMI-1640, resuspended in complete medium then transferred (7.5 × 10^5^ cells/well, in triplicate) to an ELISA MaxiSorp Nunc plate previously coated overnight at 4 °C with the anti-p17 mAb MBS-15 (10 μg/ml in PBS). After a 16 h-incubation, cells were removed and biotinylated anti-p17 mAb MBS-34 (1 μg/ml) was added to the wells for 1 h at 37 °C. Detection of immunocomplexes was performed using a peroxidase-labelled streptavidin (BD Biosciences) and 3,3,5,5-tetramethylbenzidine (Sigma) as chromogenic substrate. A standard curve for p17 quantification was generated by serial dilution of recombinant p17 (0.25–18 nM) and the amount of secreted p17 was calculated by linear interpolation. In some experiments, nucleofected cells were treated with neomycin [as PI(4,5)P_2_ inhibitor] (Life Technologies, Monza, Italy), with a protease inhibitor cocktail (Sigma) containing pepstatin-A (an inhibitor of aspartyl proteases), aprotinin (a serine protease inhibitor), bestatin (an aminopeptidase inhibitor) and leupeptin (an inhibitor of serine and cysteine protease) (Sigma), with each single component of the protease inhibitor cocktail (all purchased by Sigma), and with specific inhibitors of γ-secretase, namely LY411575 and Avagacestat (Cayman Chemical, Ann Arbor, MI).

### Cell viability assay

Cells were labelled before nucleofection with carboxy-fluorescein diacetate succinimidyl ester (CFSE) in accordance with the manufacturer’s instructions (CellTrace™ CFSE Cell Proliferation Kit, Invitrogen, Carlsbad, CA). Briefly, cells were resuspended at a density of 10^6^ cell/ml in PBS with 0.1% bovine serum albumin (BSA, AppliChem, Missouri, USA) and CFSE was added at a final concentration of 2.5 μM. After 10 min, the unbound CFSE was quenched by the addition of ice-cold complete medium (5/1 vol/vol) and by two additional washings. Cellular lysate and supernatants derived from ELISA plates were tested for CSFE content by fluorometric technique.

### p17 immunoprecipitation and Western Blot

Cells (1.2 × 10^7^) were nucleofected with AG49CMV*Gag*-RTEm26CTE as above. After 48 h, cells and supernatants were harvested and subjected to p17 immunoprecipitation. Cells were lysed in 200 μL of lysis buffer [10 mM Hepes (pH7.9), 10 mM KCl, 1.5 mM MgCl_2_, 0.5 mM EGTA, 0.5 mM EDTA, 0.6% Nonidet P-40] containing a mixture of protease inhibitors (Complete Mini; Roche) and protein concentration was determined using BCA protein assay kit (Pierce, Rockford, IL, USA). Cell supernatants were concentrated by Amicon® Ultra-4 Centrifugal Filter Units (3 kDa) (Millipore, Darmstadt, Germany). Immunoprecipitation was performed using a rabbit polyclonal antibodies to p17 (PRIMM, Milan, Italy) and the Protein G PLUS-Agarose Immunoprecipitation Reagent (Santa Cruz Biotechnology, CA, USA) following the manufacturer’s suggestion. Immunoprecipitated proteins were then detected by Western blotting using mAb MBS-3 to p17 and peroxidase-conjugated goat anti-mouse IgG (Thermo Scientific, Waltham, MA). The immune complex was revealed using the ECL System (Santa Cruz Biotechnology, Dallas, TX).

### Wound Healing Assay

HUVECs were plated into 24-well plates (1 × 10^5^cells/well) in EGM containing 10% FCS and confluent monolayers were starved for 24 h by replacing medium with EGM containing 0,5% FCS. HUVECs were then co-cultured with Jurkat cells previously nucleofected or not with AG49CMV*Gag*-RTEm26CTE (Jurkat-*Gag*) and pEGFPN3 (Jurkat-*EGFP*). After 24 h of culture, Jurkat cells were removed and HUVEC monolayers were scratched using a 200 μL pipette tip. In some experiments starved HUVEC monolayers were scratched, washed with warm PBS and cultured in EGM containing 10% FCS in the presence or absence of synthetic p17s at different concentrations. HUVEC migration was evaluated at different time points using an inverted microscope (DM-IRB microscope system, Leica, Milan Italy). HUVECs migrating into the wounded area, or protruding from the border of the wound, were photographed using a CCD camera (Hitachi Inc., Krefeld, Germany) connected to a computer via a frame grabber (Matrox Meteor). Analysis of the images were performed using the QWin-lite software (Leica).

### Confocal microscopy

HeLa cells were nucleofected with AG49CMV*Gag*-RTEm26CTE (HeLa-*Gag*), cultured for 24 h in complete medium and then fixed with 3% paraformaldehyde/2% sucrose in PBS for 30 min, permeabilized with 0.5% Tween 20, and saturated with 1% BSA in PBS. For staining, the cells were incubated for 1 h with the anti-p17 mAb MBS-3 followed by Alexa Fluor 488-conjugated anti-mouse IgG (Molecular Probes, Eugene, OR). Nuclei were counterstained with 4′,6-diamidino,2-phenylindole (DAPI, Sigma). In some experiments, HeLa cells were nucleofected with AG49CMV*Gag*K26T/K27T-RTEm26CTE (HeLa-*Gag*K26T/K27T), a construct carrying K26T and K27T mutations in the *Gag* sequence, AG49CMV*Gag*K30T/K32T-RTEm26CTE (HeLa-*Gag*K30T/K32T), a construct carrying K30T and K32T mutations in the *Gag* sequence, AG49CMV*Gag*W36A-RTEm26CTE (HeLa-*Gag*W36A), a construct carrying a W36A mutation in the *Gag* sequence and with AG49CMV*p17*-RTEm26CTE (HeLa-*p17*) and AG49CMV*p17*G2A-RTEm26CTE (HeLa-*p17*G2A), two constructs carrying myr^+ ^ and myr^−^ p17, respectively^65^. HeLa-*Gag* were also treated with neomycin [as PI(4,5)P_2_ inhibitor] (Life Technologies, Monza, Italy), with a protease inhibitor cocktail (Sigma), and with the specific cellular aspartyl protease inhibitor Pepstatin A (Sigma). In some experiments, HeLa cells were co-nucleofected with AG49CMV*Gag*-RTEm26CTE and the 5ptaseIV expression plasmid pcDNA4TO/Myc5ptaseIV^66^ or with AG49CMV*Gag*-RTEm26CTE and the mutant expression plasmid pcDNA4TO/Myc Δ15ptaseIV^40^. Cells were analyzed using a Zeiss LSM510 confocal microscope equipped with a Plan-Apochromat 63x/1.4 NA oil objective. Orthogonal z-stack sections reconstitution were obtained through AxioVision Inside 4D module.

### Peptide synthesis

A peptide (H_3_COC-SNQVSQNYPIVQNLQGQ-CONH_2_) containing the HIV-1 protease specific cleavage site (PI) between matrix protein p17 and core protein p24 (p17/p24) was synthesized using FMOC-protected amino acid (Flamma, Bergamo, Italy) by solid-phase chemistry on a Rink Amide resin and used as a substrate for different enzymes. Synthesis were carried out using an Applied Biosystems 433 A peptide synthesizer (Life Technologies, Monza, Italy) at 0.1 mM scale. Peptide was purified by reverse phase HPLC and characterized by matrix-assisted laser desorption/ionization -time-of-flight (MALDI-TOF) mass spectrometry (MS) analysis, using a Bruker Reflex III TOF mass spectrometer operating in reflector mode (Bruker, Billerica, MA, USA). The purity was always >95%.

### Preparation of enzyme and p17/p24 peptide solutions

Three μl of the stock solution of biologically active HIV-1 protease (Abcam, Cambridge, UK) (200 ng/μl, 25.59 μM) were diluted with HIV-1 protease solution (20% Glycerol, Tris-HCl 20 mM, KCl 500 mM, DTT 1 mM, EDTA 200 μM, pH 8) to obtain a final working solutions of 30 μl containing 100 ng of HIV-1 protease. Three μl of Renin (Anaspec, San Jose, CA, USA) stock solution of 50 ng/μl were diluted with 3-(*N*-morpholino) propanesulfonic acid (Mops) 50 mM buffer, pH 7, to obtain a final working solution of 30 μl (18 ng). Lyophilized Cathepsin D (Sigma) was dissolved in double distilled water (ddH_2_O) to obtain a final working solution of 30 μl (100 ng). Stock solution of 50 μM of BACE1 (Sigma) was diluted in acetate buffer 50 mM, pH 4.5, to obtain a final working concentration of 420 pM in 30 μl. Aliquots of 100 μg of p17-p24 were solubilized in DMSO to obtain a concentration of 100 μM. This solution was then further diluted in acetate buffer 50 mM, pH 4.5, or in ddH_2_O to obtain a standard working solution of 6.25 μM.

### Enzymatic Cleavage and Mass Spectrometry Analysis

Twenty μl of standard working solution of p17-p24 [dissolved in H_2_O for Renin, Cathepsin D and HIV-1 protease or in acetate buffer (50 mM, pH 4.5) for BACE1] were added and incubated at 37 °C for 30 min. The determination of p17-p24 cleavage sites was carried out by MALDI-TOF. Briefly, 1 μl of sample was mixed with 1 μl of HCCA matrix [a saturated solution of α-cyano-4-hydroxycinnamic acid (Sigma) in 50/50/0.1% of water/acetonitrile/trifluoroacetic acid] and spotted on metal target plate (MTP 384 Ground Steel, Bruker). Mass spectra were acquired with 200 laser shots for sample at a constant laser energy in reflector mode and elaborate using Flex Analysis software.

### Protein synthesis

Chemical synthesis of p17 and of a C-terminally truncated forms of p17 lacking the last 17 amino acids (p17Δ17) via native chemical ligation was performed as described previously^67^. Synthetic proteins, after HPLC purification to homogeneity, were folded by dissolving lyophilized polypeptide at 1 mg/ml in 0.2 M phosphate buffer saline (PBS) containing 6 M GuHCl, pH 7.4, followed by a six-fold dilution with 0.2 M PBS containing 10 mg/ml DTT and extensive dialysis against 0.2 M PBS. Proteins were quantified by UV absorbance measurements at 280 nm using calculated molar extinction coefficients as described^68^.

### Nucleic magnetic resonance (NMR)

NMR spectra were recorded at 25 °C on an 800 MHz (800.27 MHz for protons) Bruker Avance-series NMR spectrometer equipped with four frequency channels and a 5 mm triple-resonance z-axis gradient cryogenic probehead. A one-second relaxation delay was used, and quadrature detection in the indirect dimensions was obtained with states-TPPI phase cycling; initial delays in the indirect dimensions were set to give zero- and first-order phase corrections of 90° and –180°, respectively^69^,^70^. Data were processed using nmrPipe on Mac OS X workstations. 2D NOESY experiments with a 150 ms mixing time were collected to monitor changes in the backbone and side-chain 1 H protein resonances^71^. Typical NMR samples contained 3 mg/ml protein in a 10 mM sodium phosphate and 25 mM sodium chloride buffer, pH 7.4, to which 10% D2O (v/v) was added.

### Statistical Analysis

Data obtained from multiple independent experiments are expressed as the mean ± SD. Data were analyzed for statistical significance using the Student’s two-tailed *t* test or one-way ANOVA, when appropriate. Bonferroni’s post-test was used to compare data. Differences were considered significant at *P* < 0.05. Statistical tests were performed using GraphPad Prism 5 software.

## Additional Information

**How to cite this article**: Caccuri, F. *et al*. Cellular aspartyl proteases promote the unconventional secretion of biologically active HIV-1 matrix protein p17. *Sci. Rep.* **6**, 38027; doi: 10.1038/srep38027 (2016).

**Publisher's note:** Springer Nature remains neutral with regard to jurisdictional claims in published maps and institutional affiliations.

## Supplementary Material

Supplementary Information
