# Supplementary material for: Cellular aspartyl proteases promote the unconventional secretion of biologically active HIV-1 matrix protein p17
Source: Sci Rep. 2016 Dec 1;6:38027. doi: 10.1038/srep38027 (PMC5131311; doi:10.1038/srep38027)
Supplement: Supplementary Information [file srep38027-s1.pdf]

Cellular aspartyl proteases promote the unconventional secretion of biologically active HIV-1 matrix protein p17.

Francesca Caccuri, Maria Luisa Iaria, Federica Campilongo, Kristen Varney, Alessandro Rossi, Stefania Mitola, Silvia Schiarea, Antonella Bugatti, Pietro Mazzuca, Cinzia Giagulli, Simona Fiorentini, Wuyuan Lu, Mario Salmona, Arnaldo Caruso.

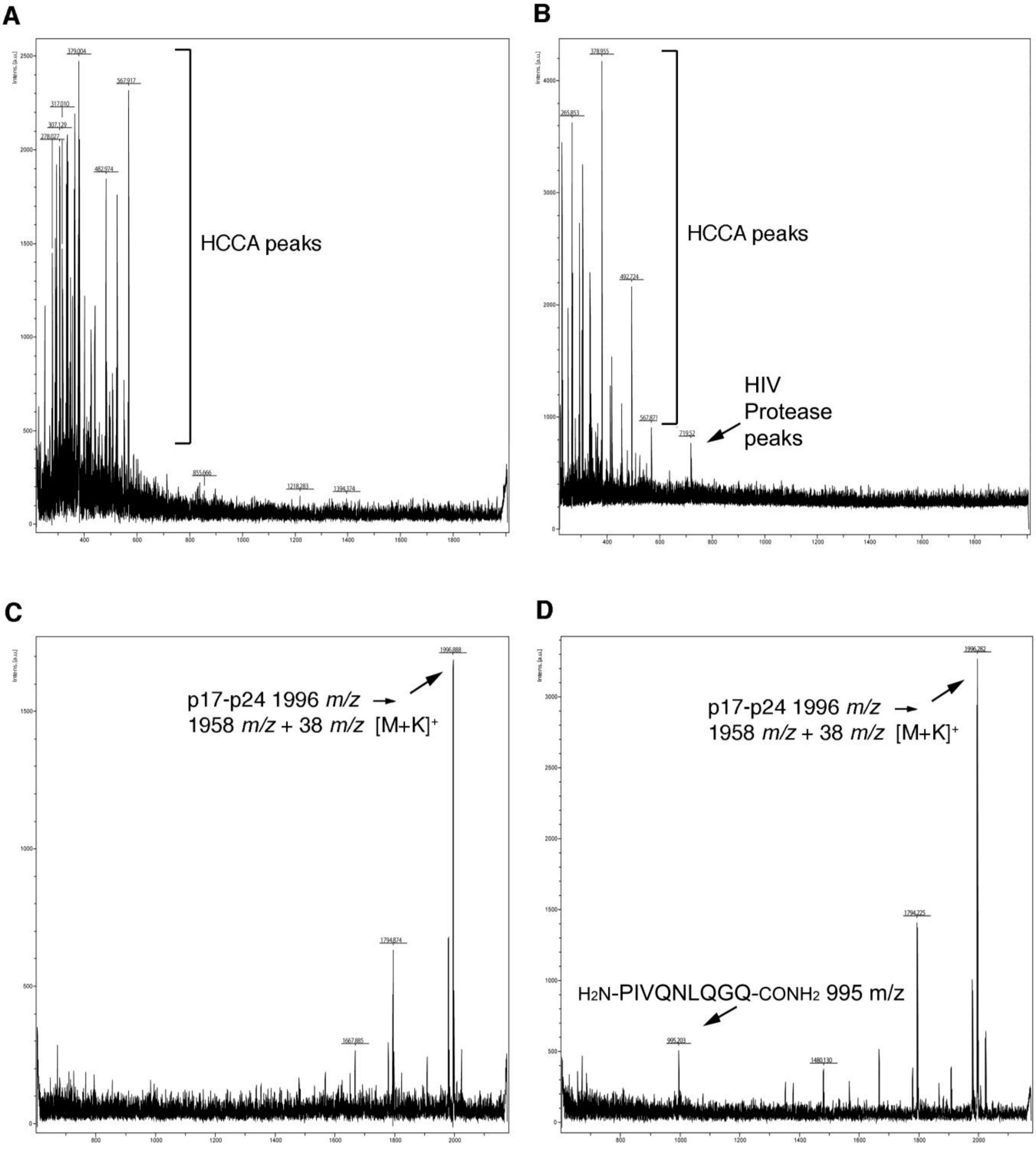

Figure S1. Cleavage of p17-p24 by HIV-1 protease. Samples were spotted on a metal plate (MTP 384 Ground Steel, Bruker) and analyzed by MALDI-TOF mass spectrometry. **A**: HCCA; **B**: HIV Protease; **C**: p17-p24; **D**: p17-p24 6.25 µM + HIV-1 Protease 100 ng after 30 min of incubation at 37°C.

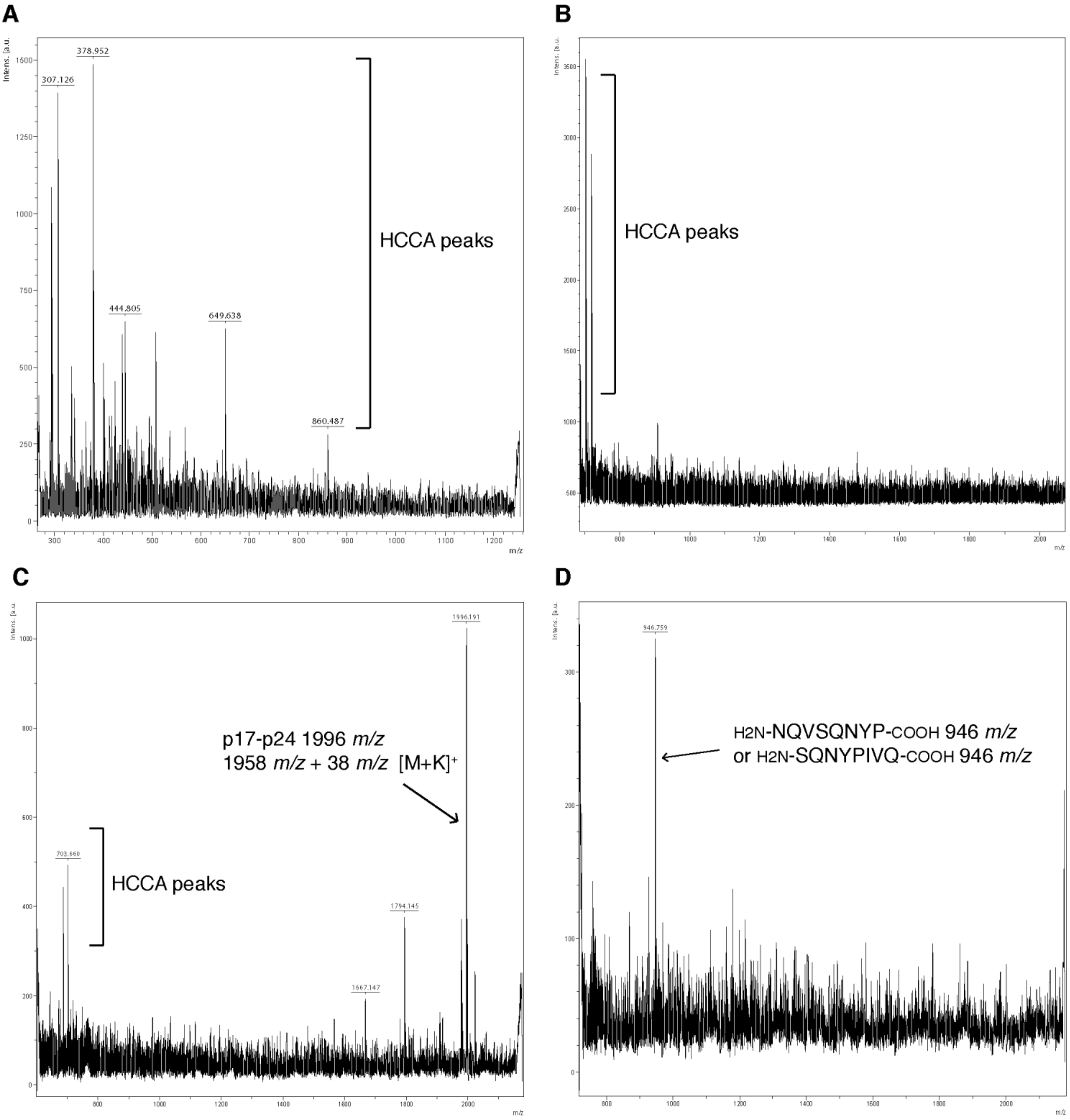

Figure S2. Cleavage of p17-p24 by Renin. Samples were spotted on a metal plate (MTP 384 Ground Steel, Bruker) and analyzed by MALDI-TOF mass spectrometry. **A**: HCCA; **B**: Renin; **C**: p17-p24; **D**: p17-p24 6.25 µM + Renin 18 ng after 30 min of incubation at 37°C.

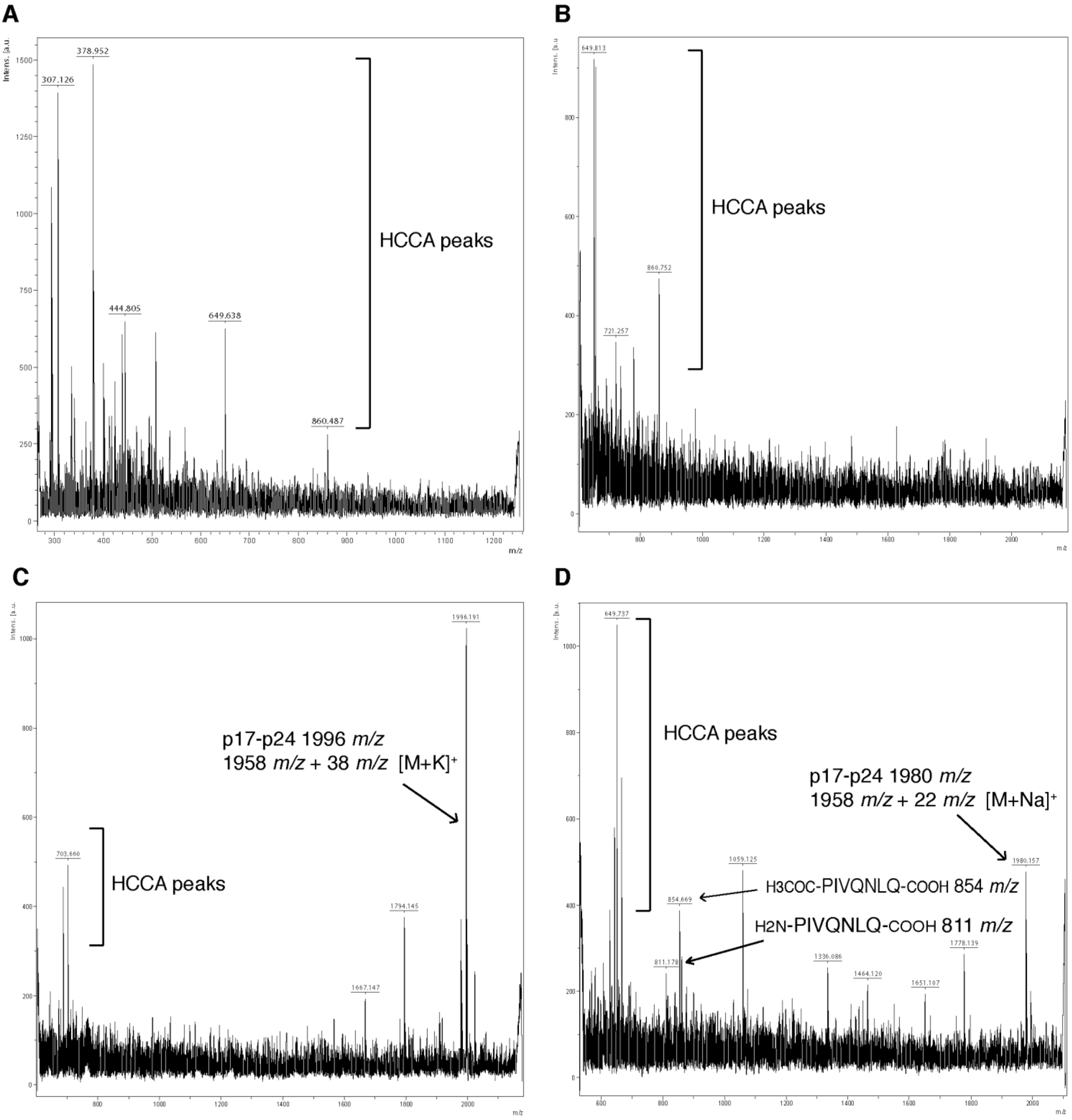

Figure S3. Cleavage of p17-p24 by Cathepsin D. Samples were spotted on a metal plate and analyzed by MALDI-TOF mass spectrometry. **A**: HCCA; **B**: Cathepsin D; **C**: p17-p24; **D**: p17-p24 6.25 µM + Cathepsin D 100 ng after 30 min of incubation at 37°C.

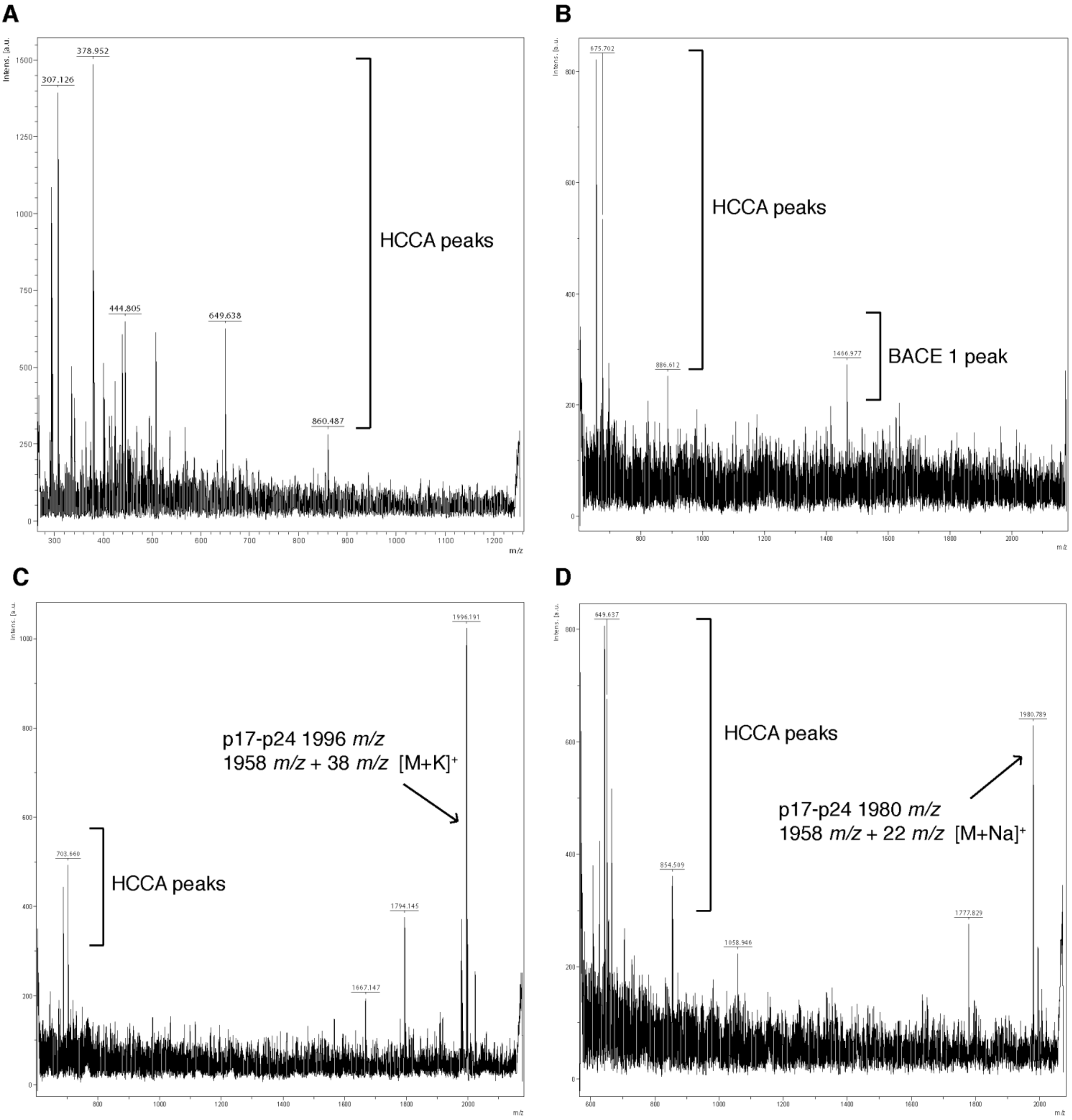

Figure S4. Cleavage of p17-p24 by BACE 1. Samples were spotted on a metal plate and analyzed by MALDI-TOF mass spectrometry. **A**: HCCA; **B**: BACE 1; **C**: p17-p24; **D**: p17-p24 6.25 µM + BACE 1 420 pM after 30 min of incubation at 37°C.

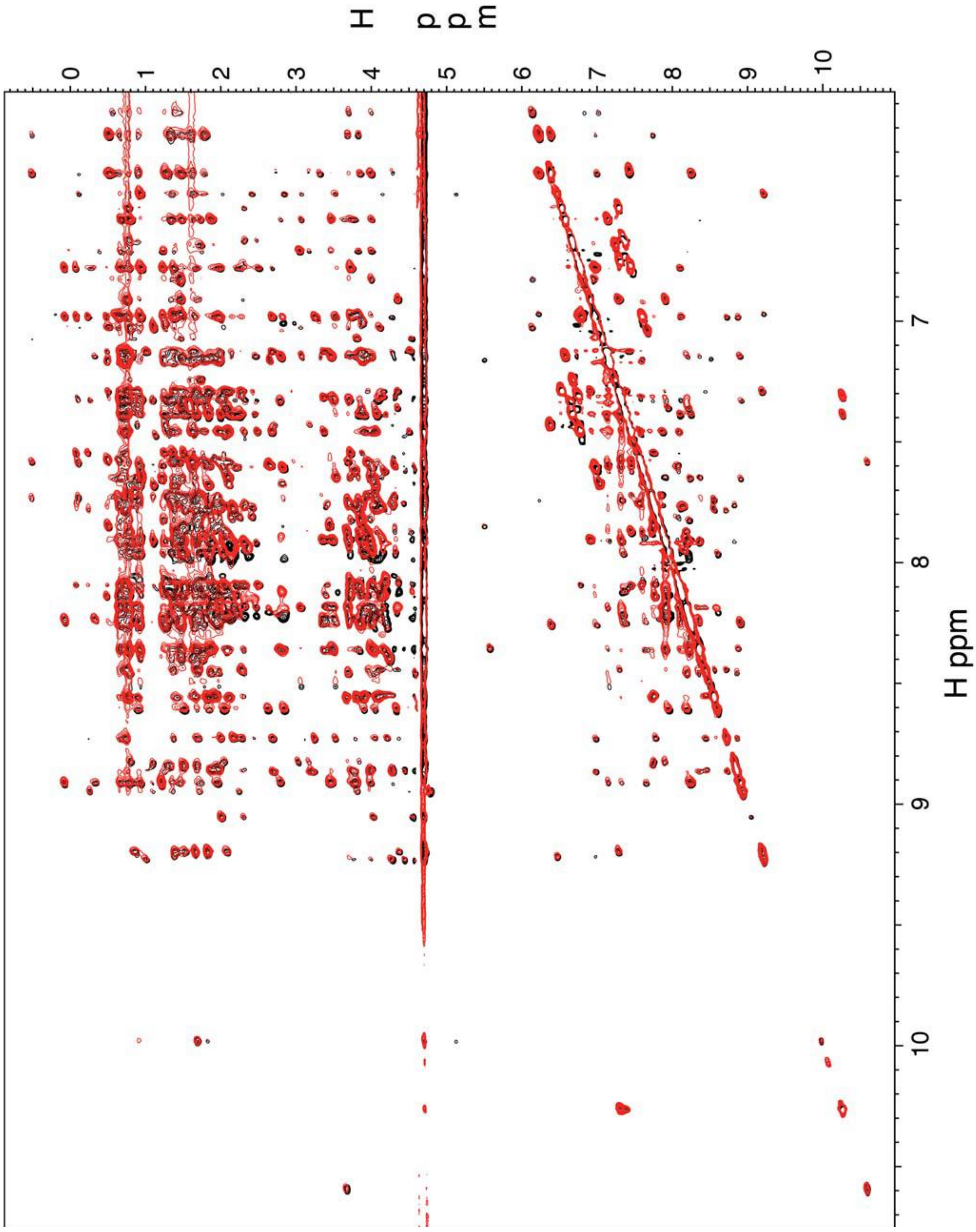

Figure S5. Two-dimensional NMR spectroscopic analysis of recombinant refp17 (black) and synthetic (2-115) refp17 (red). Signals of H^{α}-H^{N} (aliphatic-amide) nuclear Overhauser effects (NOEs) are shown on the left, and H^{N}-H^{N} (amide-amide) NOEs on the right. Nearly all H^{N}-H^{N} cross signals of synthetic (2-115) refp17 (red, right) are superimposable with those of recombinant refp17 (black, right), suggesting that the structures of the two proteins are highly similar, if not identical.
